# Expert-recommended tasks for hospital librarians during a healthcare system merger or acquisition: an e-Delphi consensus statement

**DOI:** 10.5195/jmla.2026.2031

**Published:** 2026-04-01

**Authors:** Stacy Posillico, Jaclyn Morales, Saori Wendy Herman

**Affiliations:** 1 sposillico@northwell.edu, Senior Librarian, Eastern Region Hospitals of Northwell Health, Office of Academic Affairs, Northwell, New Hyde Park, NY and Office of Academic Affairs, New Hyde Park, NY; 2 jmorales31@northwell.edu, Senior Librarian, Northwell, New Hyde Park, NY and North Shore University Hospital, Manhasset, NY; 3 saori.w.herman@hofstra.edu, Assistant Dean of Library Services, Donald and Barbara Zucker School of Medicine at Hofstra/Northwell, Hempstead, NY, and Corporate Director of Libraries, Northwell Health, New Hyde Park, NY

**Keywords:** Delphi method, Mergers, acquisitions, hospital libraries, hospital librarianship, hospital librarians, M&A, mergers and acquisitions, Consensus, leadership, management, administration, change management, Organizational Change

## Abstract

**Objective:**

Limited empirical research is available to guide hospital librarians through a healthcare system merger or acquisition. To address this knowledge gap, an e-Delphi research study was used to develop recommended tasks that librarians should consider when consolidating the delivery of library services to a newly merged, geographically distributed healthcare system.

**Methods:**

This e-Delphi study was conducted and reported according to the Guidance on Conducting and REporting DElphi Studies (CREDES). The expert panel, composed of 29 hospital librarians, responded to four rounds of questionnaires during April to December 2022. In Round 1, the panelists' qualitative responses were collected and analyzed via thematic analysis to identify potential recommended tasks. In Rounds 2 through 4, tasks were eliminated or prioritized based upon the panelists' rating of each task using a seven-point Likert scale. Those tasks rated as 5, 6, or 7 by ≥75% of the panelists were included in the final consensus statement.

**Results:**

The consensus statement identifies 330 recommended tasks. Highly prioritized tasks involve cultivating beneficial relationships with others throughout the merger, particularly newly blended library teams, finance and administrative leadership, information technology/services, and vendors. Marketing and outreach activities and physical library space management tasks were not prioritized. The panelists emphasized understanding organizational context and culture throughout any merger.

**Conclusions:**

The recommended tasks can be used by hospital librarians to create an action plan for consolidating and delivering library services in the event of a healthcare system merger or acquisition. Future research on the utility of the recommendations is anticipated.

## INTRODUCTION

It is often an isolating, disorienting experience: that moment when a hospital librarian learns their hospital or healthcare system is merging with another system or being acquired by another hospital [1–6]. Sometimes, the new addition to the system is nearby; other times the merger occurs between hospitals at a great distance from one another, both in miles and in organizational culture [4–11]. The story unfolds as the hospital librarian navigates the merger or acquisition with minimal structure to guide them [1–17]. Along the way, the hospital librarian tenaciously works to adjust and adapt the delivery of library resources and services over the months or years it takes for the hospitals to fuse into one newly reconstituted organization [1–17].

Medical librarians regularly manage the delivery of services to multiple geographically dispersed hospitals, and this responsibility has long been established as a core aspect of their role [2, 6, 8, 18]. However, since 2012, hospital librarians in the United States (U.S.) are increasingly required to navigate their libraries through the rising number of healthcare systems and hospital mergers and acquisitions [5, 6, 14, 19].

The COVID-19 pandemic briefly slowed down the need for hospital librarians to respond to the steady procession of merger activity [20]. By the end of 2023, however, this expansion by consolidation was on the rise again and is expected to continue [19, 21, 22], “leading the nation to a point where there will soon be very few small healthcare organizations left” [23].

Hospital librarians are faced with the continuing challenge of how to serve the information and education needs of library users within larger U.S. healthcare systems [24] that care for diverse patient communities, sometimes at a great distance from each other [19, 25–31]. For example, as academic medical centers are increasingly acquiring community hospitals for the talent and financial benefits they provide [19, 21, 22], hospital librarians must elevate their own skills in order to meaningfully contribute to advancements in the rigor and impact of scholarly activities within the growing healthcare system.

Hospital librarians may be required to advance their own education and knowledge as the clinical work at the newly merged system shifts and develops greater specialization and complexity [2, 3, 6, 8]. At the same time, hospital librarians must educate those community hospital clinicians who have little experience with access to library resources or assistance from librarians about the full range of services librarians provide [1, 2, 3, 7, 8, 17].

Throughout the merger process, hospital library budgets may be diminished by any reductions and eliminations needed to attain financial benefits for the merging system [23, 28–33]. Physical library spaces can be lost to new or reorganized staff that need space, particularly in healthcare systems where library users have mostly moved to virtual workspaces and using online information resources [34, 35]. Hospital librarians have long struggled with promoting and marketing their availability and value [35–37]. Once involved in a merger, they need to find new and creative ways to reassure current library users that the quality of services will be maintained and to raise awareness of their existence with newcomers to the system [7, 11, 33, 34, 37].

Coinciding with these challenges, little empirical research is available for librarians to use in these situations. In contrast to the well-researched and debated guidelines, legal requirements, and ethical standards available to hospital executives during a merger or acquisition [12, 21, 28, 32, 38–40], an extensive literature review revealed that there are no research-based models or validated guidance for how to consolidate, collaborate, and deliver library services across institutions that may be miles or states apart and differ wildly in culture and custom, despite the fact that medical librarians have called for such research [1, 3–5, 7, 8, 12, 13], The literature on this topic is found primarily in case studies or reports of the experience by hospital librarians who have been there and shared their experience in hopes that it will benefit a colleague faced with the same difficult choices and challenges to create a newly merged hospital library [1–17, 41].

This method of reconciling services and resources based on limited reporting in the literature can lead hospital librarians to make decisions that may not fully integrate library services and resources across the newly formed institution [3, 5, 7, 12–15, 41]. This can lead hospital libraries to forego the positive effects of full financial and cultural integration [28], although integration that prioritizes centralization has been shown to provide the most benefit to hospitals, staff, patients, and the communities served by a newly merged system [21, 23].

Because there is limited empirical research available to guide hospital librarians through a healthcare system merger or acquisition, the purpose of this modified e-Delphi study seeks to address this knowledge gap and create evidence-based recommendations for providing access to information resources and delivering hospital library services through one comprehensive, geographically distributed system following a healthcare system merger or acquisition.

## METHODS

### Justification for e-Delphi Research Method

This study utilized a modified electronic Delphi (e-Delphi) method to develop a consensus statement of recommended tasks (referred to as “consensus statement”) [42–46]. This iterative process involves anonymous surveys emailed to experts to reach consensus [42, 43, 46, 47]. Given the complexity of hospital library mergers and acquisitions and the divergent literature [42, 43, 48, 49], the e-Delphi method allowed for a comprehensive understanding of hospital librarians' priorities [42, 43, 46, 47, 49–52] during mergers or acquisitions.

Experimental methods were impractical due to challenges in recruiting sample and control groups and the lack of a testable intervention [53]. The e-Delphi technique mitigated the limitations of the survey [46, 50] and interview methods where individual opinion is solicited without consensus. It also avoided the limitations of the focus group where psychological interference can lead to the potential for few individuals to overtake the group, thus suppressing valid opinion.

### Study Design and Protocol

This research study was conducted and reported according to the Guidance on Conducting and REporting DElphi Studies (CREDES) [42]. An e-Delphi research protocol was designed that included: 1) a group of panelists that have clearly identified leadership experience with a merger or acquisition; 2) multiple rounds of questioning the panelists, allowing them to refine their opinion as the process proceeded; 3) anonymity among the panelists, so they could freely express opinion without the social pressures of conformity or fear that their responses would be made public to their employers; and 4) statistical analysis of the final group response, having reached a level of consensus that was pre-defined by the researchers [42, 43, 45, 46].

Prior to designing the research protocol, a thorough literature search was conducted to identify published literature about hospital librarians’ experiences in a healthcare system merger or acquisition. Based on the results of the literature search, a preliminary qualitative questionnaire was created.

To establish content and face validity [56, 57], the research team invited nine medical librarians who did not fully meet the pre-established expert panelist criteria but had experience with a hospital merger or acquisition or the Delphi method, to pilot the Round 1 questionnaire. The anonymous feedback received via a SurveyMonkey evaluation form was incorporated into the final Round 1 questionnaire. The pilot respondents validated the questionnaire but also suggested that definitions of specific terms and examples of tasks be added as an optional preview to minimize bias and to provide clarity [45].

The research team deviated from the classic Delphi mail approach in order to efficiently communicate with the panelists [42, 43] and used a web-based method for collecting responses. All the questionnaires were created and distributed via a secure Internet-based survey using the Research Electronic Data Capture (REDCap) system managed by Northwell Health [58]. Responses to all the questionnaires received via REDCap were anonymized to the researchers, and the panelists were never identified to each other.

In planning the study, the research team anticipated a large dataset being produced from Round 1, which would require considerable and careful analysis to process the data into the Round 2 questionnaire. As a result, a research assistant was recruited to help with data organization and management. A research grant of $1,000.00 was awarded from The Liberty Chapter of the Medical Library Association in October 2021 and combined with funding from Hofstra University’s Stuart and Nancy Rabinowitz Honors College Undergraduate Research Assistant program to support the research assistant’s work over the course of three academic semesters in 2022. This research study received approval from the Institutional Review Board (IRB) of Northwell Health on January 18, 2022, as an exempt research study (Study ID No. 21-1336-EXT).

### Panel Recruitment

The pre-established criteria for participating in this research study required that all accepted panelists have the prerequisite experience [43, 46, 50, 53] of being a hospital library professional who had either led or been actively involved in a non-leadership role during a U.S.-based hospital or healthcare system merger beginning 2010 through the date of recruitment. To ensure a geographically diverse panel, the researchers sought to empanel at least one participant from each of the seven regions of the Network of the National Library of Medicine (NNLM) [61]. The research team planned to empanel 23 to 30 hospital librarians in the study to produce reliable work [43].

Recruitment for panelists opened nationwide in February 2022 and was conducted through targeted emails to library professionals identified through the literature search and email campaigns through the Medical Library Association’s caucus and chapter listservs. All interested persons were asked to complete a nine-question survey via SurveyMonkey. The interest form closed in March 2022. Each researcher independently assessed the de-identified responses using a rubric based on the predetermined eligibility criteria. The research team met and selected 32 prospective panelists. Twenty-nine expert panelists accepted the invitation to participate in the study. Among the accepted panelists, there were representatives from each of the seven regions of the NNLM network [61].

### Study Timeline

**Figure 1:**
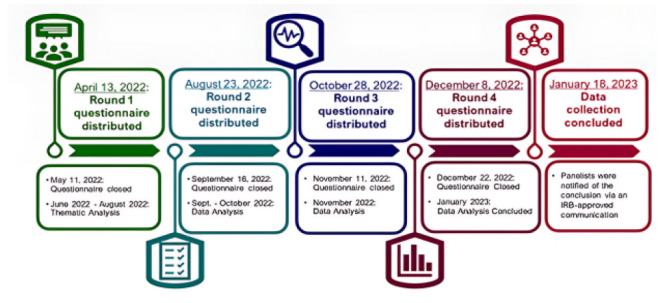
Timeline of e-Delphi Research Study Rounds

The panelists completed four rounds of questionnaires from April to December 2022. Figure 1 portrays the research study timeline.

To ensure anonymity, individual panelists' responses in each round were not tracked, leading to slight variations in the total number of responses received in each round. All four questionnaires included a closing date, and at least two email reminders were sent before closure. Short extensions were granted during each round to maximize response rates while respecting panelists' time [45].

### Round 1 of the e-Delphi Study

In Round 1, the panelists were asked to identify key tasks to be addressed during a merger. The questionnaire was divided into four sections: Tasks to be addressed prior to the merger or acquisition; tasks to be addressed regarding the hospital or healthcare system merger or acquisition; tasks to be addressed regarding centralization of library services; and questions regarding the panelists’ experience with the merger or acquisition process within a hospital library. Panelists entered in the key tasks as free text.

The data collected in Round 1 was analyzed via Braun & Clarke’s six-step process of reflexive thematic analysis [59]. The research team decided to use Braun & Clarke's reflexive thematic analysis approach for qualitative analysis to convert the open-ended responses into actionable tasks. This approach specifically allowed the researchers, all of whom are experienced medical librarians at a large academic healthcare system affiliated with a medical school, to bring their knowledge and understanding of the medical library profession to the analysis as a resource [59, 60]. Responses to each Round 1 question were divided equally amongst the researchers, and the researchers read and familiarized themselves with the assigned responses [59].

After the familiarization phase, codes were generated independently by each researcher via critical reflection on their own past librarian experiences and cultural position in this profession [59]. The team regrouped to consolidate the hundreds of generated codes into action-oriented tasks [59]. Employing a constructionist thematic analysis, the research team used both semantic and latent content analysis to create a list of action-oriented tasks [59].

### Rounds 2 Through 4 of the e-Delphi Study

**Figure 2:**
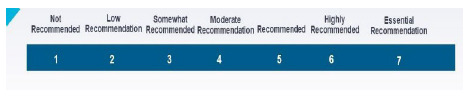
Likert Scale Used by Panelists in Rounds 2 to 4

After the thematic analysis was conducted, the research team used its familiarity with the hospital librarian profession to reorganize the action-oriented tasks from the initial four sections into 15 categories for the Rounds 2-4 questionnaires. A 7-point Likert Scale, provided in Figure 2, was utilized by the panelists to reach consensus.

Each questionnaire was approved by the IRB as a modification to the exempt research study before distribution. The researchers consulted with two statisticians on all quantitative analysis processes used during and after these three rounds.

A consensus threshold of ≥75% was determined prior to data collection based on the literature review. The tasks rated as 5, 6, or 7 by ≥75% of the panelists at the conclusion of each round are included in the final consensus statement. The tasks rated as 1 by ≥75% of the panelists at the conclusion of each round were automatically removed from future questionnaires and were not included in the final consensus statement. Any remaining tasks that did not meet the ≥75% threshold were placed in the subsequent questionnaire for a second review. If a task did not reach consensus for inclusion in the consensus statement after the second review, the item dropped out of further consideration.

Although controlled feedback is traditionally provided to panelists after each round during a Delphi study [42], aggregated results and trends were not reported to the panelists after each round in this study. By withholding accumulated results after Rounds 2 and 3, the researchers aimed to minimize social desirability bias and reduce the pressure panelists might feel to adjust their responses to align with the group rather than express their individual evaluations of the identified tasks [43]. Moreover, the considerable number of categories and individual tasks listed within each of them warranted providing the panelists with an opportunity to conduct an impartial second review in a subsequent round.

The protocol initially planned for only three rounds of the e-Delphi process, but a technical issue in REDCap removed the Collection Development (29 tasks) and Vendor Relations (29 tasks) categories from the Round 2 review. Panelists first reviewed these two categories in Round 3, after completing their second review of the other 13 categories. Round 4 consisted of the second review for only those two categories.

Furthermore, the Round 4 questionnaire included an optional open-ended comments section for the participants, allowing them an opportunity to provide future users of the consensus statement with informed commentary that would be beneficial to consider. The comments collected in this section were assessed via the same reflexive thematic analysis processes used for the Round 1 response analysis [59].

## RESULTS

A total of 29 panelists had the opportunity to participate in all four rounds; however, not everyone completed each questionnaire by the specified deadline. In consultation with a biostatistician, the slight variation in the number of participants for each round did not affect the statistical reliability of our results.

**Figure 3:**
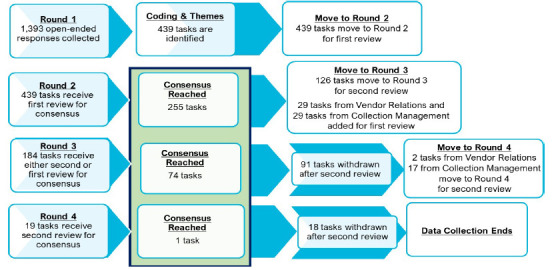
Consensus Reached in Each Round

Over the course of four rounds, 1,393 open-ended responses were developed into a consensus statement of 330 recommended tasks. The consensus reached in each of the rounds is described in Figure 3.

### Round 1

Twenty-five of the 29 panelists (86%) completed the questionnaire, and a total of 1,393 open-ended responses were collected. After thematic analysis, the 1,393 responses were developed into lists of action-oriented tasks. The research team identified 439 tasks and thematically organized the tasks into 15 categories for the panelists to consider in the Round 2 questionnaire. The categories that had the largest number of recommended tasks to consider were: Interpersonal Wellness; Library Policies, Procedures, and Job Responsibilities; and Information Technology and Services. The categories that had the smallest number of tasks to consider were: Physical Library Spaces, System Organizational Structure, and Library Staff Training and Development.

### Round 2

Twenty-two panelists completed the Round 2 questionnaire. Two panelists formally withdrew from the study after the Round 2 questionnaire was distributed. A total of 255 tasks from 13 categories reached consensus as recommended by at least 75% of the panelists’ rating the task as 5 (“Recommended”), 6 (“Highly Recommended”) or 7 (“Essential Recommendation”) on the Likert scale. None of the tasks reviewed received a consensus rating of 1 (“Not Recommended”) by 75% or more of the panelists. A total of 126 tasks from the 13 categories did not reach consensus and were moved to the Round 3 questionnaire for a second review by the panelists.

### Round 3

Twenty-three panelists completed the Round 3 questionnaire, which asked them to review task lists for all fifteen categories. None of the tasks reviewed in any category received a consensus rating of 1 (“Not Recommended”) by 75% or more of the panelists. The panelists’ second review in 13 categories led to an additional 35 tasks reaching consensus and inclusion in the consensus statement. Ninety-one tasks from 13 categories were eliminated because they did not reach consensus at the end of this second review. Fifty-eight tasks in the Collection Management and Vendor Relations categories received their first review during this round. Twenty-seven of the 29 tasks from the Vendor Relations category reached consensus, and two tasks moved over to the Round 4 questionnaire. Twelve tasks from the Collection Management category reached consensus, and 17 moved over to the Round 4 questionnaire.

### Round 4

Twenty-four panelists completed the Round 4 questionnaire. Only one task from the Collection Management category was added to the consensus statement, with the remaining 18 tasks from the two categories eliminated. None of the tasks reviewed received a consensus rating of 1 (“Not Recommended”) by 75% or more of the panelists.

Additionally, 18 open-ended responses were collected from the 24 panelists to capture any insight about the merger or acquisition process that was not reflected in the list of recommended tasks. These responses were analyzed using the same reflexive thematic analysis used for Round 1. The theme of understanding organizational context and culture throughout the merger process was recognized in many of the responses, and the researchers noted that this theme was incredibly important to the panelists.

Additionally, partnering with administration and leadership was noted as an important theme to consider when using the consensus statement. The theme of finding value in digitizing the collection and eliminating print holdings was also identified.

### The Consensus Statement of Recommended Tasks

From the 1,393 open-ended responses received in Round 1 and the 439 tasks reviewed by the panelists in Rounds 2 through 4 questionnaires, there are 330 action-oriented tasks presented in the final consensus statement. The consensus statement is provided in full in Appendix A. It is also available online at: https://libguides.hofstra.edu/ConsensusRecsHSLMA.

**Figure 4:**
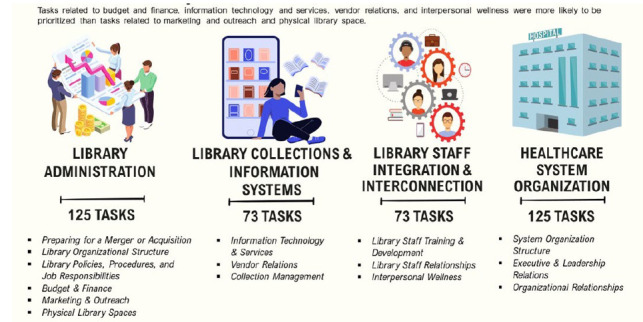
Summary of Consensus Statement of Recommended Tasks

The tasks were organized into four major categories: Library Administration, Library Collections and Information Systems, Library Staff Integration and Interconnection, and Healthcare Organization. Each major category was then divided into subcategories, as visualized in Figure 4.

Each of the tasks that reached consensus were assigned a level of recommendation, which was determined according to the mode for each task. A Level 1 recommended task represents an essential task (a mode of7) that a librarian should consider addressing immediately during a hospital merger or acquisition, whereas tasks assigned a Level 2 are highly recommended (a mode of 6), but not essential, to undertake during the merger process. Level 3 recommended tasks (a mode of 5) are those that may be considered at a later time. Of the 330 recommended tasks, 141 (43%) were assigned to Level 1 as an essential task; 123 (37%) were assigned to Level 2 as highly recommended; and 65 (20%) were assigned to Level 3 as a recommended task.

**Figure 5:**
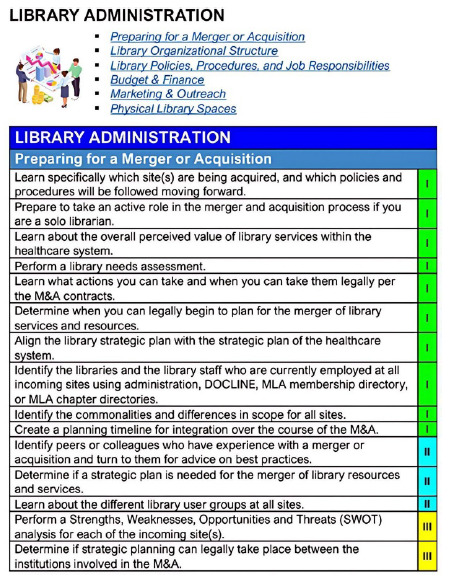
Library Administration-Preparing for a Merger or Acquisition in the Consensus Statement of Recommended Tasks

A snapshot from the main category of *Library Administration*, and a snippet of the first of its subcategories *Preparing for a Merger or Acquisition,* is provided as Figure 5 to demonstrate the look and feel of the consensus statement.

## DISCUSSION

This e-Delphi research study equips hospital librarians with the evidence-based recommendations they need to manage the challenges of a hospital or healthcare merger or acquisition, particularly as the consolidation trend continues across the United States [22, 23]. The tasks recommended by panelists include a particular focus on building collaborative relationships outside the library and nurturing library staff wellness. Additionally, they reflect the evolution of hospital libraries to online environments and underscore the importance of organizational context and culture.

### Primary Tasks Recommended by The Panelists

A review of the consensus statement reveals that, from the panelists’ experienced perspective, hospital library leaders must prioritize creating beneficial, cooperative relationships with key executives, financial team members, IT/IS departments, and vendors.

Almost all the Level I recommended tasks in the Executive & Leadership Relations subcategory pertain to creating connections with the administration that allows for productive conversations about library budgets, payment responsibilities, and cost allocations. The Budget and Finance subcategory encompasses many Level I recommended tasks that address library leaders becoming knowledgeable about budgets and finance so that they can have well-informed conversations with the library’s internal and external stakeholders.

The prioritization of these tasks reflects the ongoing discussion in our profession about maintaining sufficient budgets, especially during times of change, to continue providing library access and services to existing and incoming users [1–3, 9, 12, 14, 17, 23, 28, 33, 34, 41]. Establishing beneficial relationships with key financial decision-makers is fundamental to sustaining operational funding for the library throughout the merger process [1, 3, 7, 12, 15, 17, 33].

Creating relationships with the IT/IS department for a newly merged system was similarly at the forefront of the panelists’ recommendations. The fact that 61% (20/33) of the Information Technology and Services tasks are a Level I recommendation aligns with published literature [33, 34] that highlights the need for partnership and collaboration with the IT/IS team to maintain current service levels and to expand access to incoming sites [4, 7, 9, 16, 17].

Collection development and vendor relations are traditionally closely associated in the library nomenclature. However, the consensus statement did not reflect that. Many of the Collection Development subcategory tasks are logistical undertakings that can be quickly accomplished, while the entirety of the Vendor Relations subcategory emphasizes the need to build trustworthy and reliable relationships with vendors who provide library information resources [4, 7, 9, 12, 17, 33].

While developing relationships with external departments and vendor partners may allow the hospital library to grow during the merger or acquisition process, the Interpersonal Wellness subcategory emphasizes another priority of paramount importance to the panelists: the need to nurture the internal relationships among the librarians who will be working together as the merger progresses. This subcategory has the highest number of recommended tasks among all the subcategories and includes tasks such as:

- Ensure you are sensitive and considerate to how people adjust to change in different ways. (Level I)
- Acknowledge the frustration that comes when you and others do not feel heard during the M&A process. (Level I)
- Acknowledge the transformational nature of the change an M&A brings, and that such change takes time. (Level II)

The recommended tasks presented in this subcategory correspond closely with the published literature that explores the sometimes frightening and disorienting experience of going through a healthcare system merger or acquisition when you are a hospital librarian [2, 3, 13–15, 17]. Accordingly, the panelists provide a compelling guide for hospital librarians leading or actively involved in a merger or acquisition to address the complex array of emotions stemming from negative feelings such as fear, trepidation, insecurity, vulnerability, and stress, along with potential positive feelings of excitement, a renewed sense of purpose, or possibly optimism for the future.

Taken as a whole, the consensus statement embodies the idea that the best available action that any hospital librarian can take during a merger or acquisition is to develop and maintain healthy relationships, particularly with library colleagues, key finance team members, IT/IS department associates, and vendor representatives.

### Peripheral Tasks Recommended by The Panelists

Marketing tasks were a surprisingly low priority for the panel, possibly reflecting the long-standing challenges hospital librarians face in demonstrating their value [1, 3, 4, 36, 37]. It is curious that more marketing tasks did not make the final consensus, because as healthcare systems merge, hospital librarians must reach out to new user communities, and at the same time, reassure existing users that library access and services will not be interrupted [1, 4, 7, 11, 12, 15, 33, 37]. However, because the panelists prioritized tasks related to relationship-building in every category, this suggests that pursuing those types of tasks as outlined in the consensus statement could naturally increase the library’s visibility, discoverability, and value, potentially replacing traditional marketing efforts. Furthermore, there are recommended tasks within the consensus statement that offer opportunities for marketing and outreach campaigns to involve hospital librarians in a system’s academic and scholarly activities, given that enhanced teaching and publishing efforts are a key driver of mergers and acquisitions [1, 3, 19, 21, 22, 35, 41].

Physical library space was also not a concern for the panelists. Only ten tasks were created in this subcategory for Round 2, and only four were included in the consensus statement. This relatively low number of tasks in the smallest of all the subcategories aligns with the movement of many hospital library resources and services to the online environment [4, 7, 16, 33–35, 41]. Our analysis of the Round 4 responses further highlighted this theme. As one of the panelists stated, “[i]n our current environments, print is used less often these days, so the priorities in a merger should be merging online collections. Our mergers involved wide geographic regions, with many locations never having a library on site. The focus was to develop online collections to meet people where they were – online.”

### The Importance of Understanding Organizational Context and Culture

“The specific context and politics of your setting will influence which and whether all recommended practices should apply,” one panelist shared in Round 4. In no other area is context and culture more important than when hospital library leaders pursue opportunities to centralize library access and services.

Thirty-four of the 36 recommended tasks listed in the Executive & Leadership Relations subcategory are an “essential recommendation” (Level I) or “highly recommended” (Level II). Many of these tasks involve working with executive leadership to determine the extent to which library services will be consolidated and access to resources will be centralized as the merger proceeds. Hospital librarians should use these tasks to work towards acquiring the endorsement and support of executive leadership for full and complete integration, as similarly discussed in the published case reports [2, 4, 9, 13, 14, 17, 19, 41], which will lead to the greatest benefits for the library, its users, and the healthcare system itself [23]. As one of the panelists said in Round 4, “[m]any components addressed in the questionnaire discuss centralization of print collections, library administration, systems administration, etc. The preference and feasibility of centralizing versus independently operated library services is highly dependent on the type of arrangement between the healthcare systems…. The driving factor of course [is] the expectations of leadership for the acquiring and the acquired systems as to how much integration should occur.”

Ideally, the culture and context of the merging or acquired hospitals and healthcare systems should determine the way any recommended task is carried out. As the consensus statement is used, hospital librarians would do well to remember these words of one of the panelists from Round 4: “No two mergers, downsizing, or whatever are the same. Take the recommendations and make them work for your specific situation.”

### Limitations

This research was designed to protect the anonymity of the panelists and allow them to speak freely of their past or current employers, and thus the research team did not connect any of the responses received with any panelist’s individual identity. We have no information about the size of the hospitals or healthcare systems involved, the type or location of the hospitals involved, the healthcare resources available, or the culture, government, and politics of the state where their merger experience occurred. Without this context, at times it may be difficult for users to discern whether a task is applicable to their own merger experience, even if it is highly recommended or an essential recommendation. Furthermore, this was a United States-based study and therefore cannot be fully generalizable to hospital libraries in healthcare systems located in other countries.

## CONCLUSION

As US healthcare systems continue to pursue expansion through consolidation, this evidence-based consensus statement fills the knowledge gap and provides hospital librarians with a roadmap from their colleagues as they adjust and acclimate the delivery of library resources and services to a newly reconstituted organization. Rather than feel isolated, the consensus statement offers hospital librarians the opportunity to consult and act upon tasks recommended by the collective experience of hospital librarians who have been through the process and learned valuable lessons about how to successfully move forward.

Moreover, we look forward to future published reporting from our colleagues about their use of the consensus statement during their merger experience, as utilization will test the recommendations reached in this study, and over time, enhance this work with a kaleidoscope of perspectives beyond that of our original panelists.

## Data Availability

Data associated with this article cannot be made publicly available because they may contain personally identifiable information. Access to the data can be requested from the corresponding author and may be subject to IRB restrictions. Study instruments are available upon request.

## SUPPLEMENTAL FILES

**Appendix A:** The Consensus Statement of Recommended Tasks for Hospital Librarians Experiencing a Merger or Acquisition in Their Organization
