# Supplementary material for: Expert-recommended tasks for hospital librarians during a healthcare system merger or acquisition: an e-Delphi consensus statement
Source: J Med Libr Assoc. 2026 Apr 13;114(2):125–35. doi: 10.5195/jmla.2026.2031 (PMC13075583; doi:10.5195/jmla.2026.2031)
Supplement: Supplementary file 1 — Appendix A: The Consensus Statement of Recommended Tasks for Hospital Librarians Experiencing a Merger or Acquisition in Their Organization [file jmla-114-2-125-s01.pdf]

APPENDIX A:
The Consensus Statement of Recommended Tasks for Hospital Librarians Experiencing a Merger or Acquisition in Their Organization

DIRECTIONS: Hospital librarians are encouraged to begin reviewing recommended tasks within the major category that is most relevant when they learn of the impending or recently achieved merger, mega-merger or acquisition (referred to as "M&A"). Subsequently, they should focus on those subcategories that need urgent action depending on the merger stage and the merging organizations' values, policies, and processes.

Once within the subcategory they wish to review, hospital librarians should then use the priority ranking chart below to decide which tasks to undertake first:

Level 1 Strength of Recommendation (Denoted as I)<br>= **Essential Recommendation** (Tasks received a mode of 7 from panelists)
Level 2 Strength of Recommendation (Denoted as II)<br>= **Highly Recommended** (Tasks received a mode of 6 from panelists)
Level 3 Strength of Recommendation (Denoted as III)<br>= **Recommended** (Tasks received a mode of 5 from panelists)

Navigation to Major Categories:

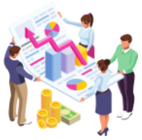

LIBRARY ADMINISTRATION

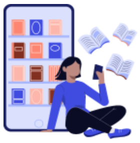

LIBRARY COLLECTIONS & INFORMATION SYSTEMS

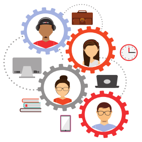

LIBRARY STAFF INTEGRATION & INTERCONNECTION

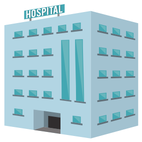

HEALTHCARE SYSTEM ORGANIZATION

LIBRARY ADMINISTRATION

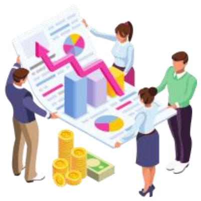

- *Preparing for a Merger or Acquisition*
- *Library Organizational Structure*
- *Library Policies, Procedures, and Job Responsibilities*
- *Budget & Finance*
- *Marketing & Outreach*
- *Physical Library Spaces*

LIBRARY ADMINISTRATION	
**Preparing for a Merger or Acquisition**	
Learn specifically which site(s) are being acquired, and which policies and procedures will be followed moving forward.	I
Prepare to take an active role in the merger and acquisition process if you are a solo librarian.	I
Learn about the overall perceived value of library services within the healthcare system.	I
Perform a library needs assessment.	I
Learn what actions you can take and when you can take them legally per the M&A contracts.	I
Determine when you can legally begin to plan for the merger of library services and resources.	I
Align the library strategic plan with the strategic plan of the healthcare system.	I
Identify the libraries and the library staff who are currently employed at all incoming sites using administration, DOCLINE, MLA membership directory, or MLA chapter directories.	I
Identify the commonalities and differences in scope for all sites.	I
Create a planning timeline for integration over the course of the M&A.	I
Identify peers or colleagues who have experience with a merger or acquisition and turn to them for advice on best practices.	II
Determine if a strategic plan is needed for the merger of library resources and services.	II
Learn about the different library user groups at all sites.	II
Perform a Strengths, Weaknesses, Opportunities and Threats (SWOT) analysis for each of the incoming site(s).	III
Determine if strategic planning can legally take place between the institutions involved in the M&A.	III

Library Organizational Structure	
Determine what the organizational structure of the library(ies) will be once the M&A is in process.	I
Determine what the reporting structure of the library(ies) will be once the M&A is in process.	I
Establish a plan to implement and communicate any reporting structure changes to the librarians and library staff.	I
Establish who is authorized to make the final decisions about contracts, costs, and library budgets in the merging library organization.	I
Identify the librarians and/or library staff that will be retained and onboarded into the merged system.	I
Identify the librarians and/or library staff positions that will be terminated during the M&A process.	I
Identify remote librarians, in-person librarians, and hybrid librarians at each site.	I
Discover if the library staff at the incoming site provide services at more than one site.	I
Determine who will manage the contracts and budgets for each library site.	I
Determine who will manage library holdings.	I
Determine who will manage access and technical services for hardware, software, web-based platforms, and e-resources.	I
Identify who will be in charge of communicating with vendors regarding technical support.	I
Determine the librarians that will manage and/or provide education and instructional services.	I
Determine who will manage the library internet/intranet presence.	I
Discover all memberships in local community resource/cost sharing groups at each site.	I
Centralize library services and resources if there are a small number of sites to accommodate future growth.	II
Determine if there can be one central library director to whom the libraries report.	II
Discover all memberships in regional consortia at each site.	III
**Library Policies, Procedures, and Job Responsibilities**	
Identify what services are currently offered at the incoming library site(s).	I
Identify the job responsibilities of the library staff from all site(s).	I
Identify any overlapping roles and responsibilities of librarians and library staff as a result of the merger, and where duplicative efforts are occurring, try to eliminate them.	I

Determine who will decide which user groups will receive library services.	I
Determine the library services that will be provided to the incoming site(s).	I
Determine the library services that will be provided to the incoming sites that do not have a library or librarian on staff.	I
Craft a unified message and a prepared script to explain to administrators and different user groups of the incoming sites why certain library services will not be offered to them.	I
Determine whether work will be distributed to library team members during and following the M&A process by hospital site, by task, or by a hybrid method of site/task.	I
Identify expectations for all librarians and library staff.	I
Explicitly describe roles and responsibilities for all library staff, librarians and volunteers.	I
Create a list of current library projects that are ongoing at each site.	II
Devise a working plan that will use all staff members to provide optimal library services, acknowledging that some roles and responsibilities will change.	II
Create a plan for the delivery and provision of library services for the incoming hospitals.	II
Focus on workflow efficiencies to address the increased size of the library user base.	II
Assess and address how geographic distance will impact library staff management and responsibilities.	II
Create staff assignments that support coverage for solo librarians at any site.	II
Identify any job title changes that will occur at each site.	II
Create a centralized system for requesting interlibrary loan and providing document delivery.	II
Decide whether to continue or deactivate DOCLINE/OCLC and make updates as needed.	II
Identify a method to gather usage statistics at each site.	II
Determine a way to get usage statistics for any site that was not previously collecting them.	II
Create a plan to consolidate library services, even if it is not known whether services will be consolidated.	III
Identify those library policies that will be followed during the M&A process.	III
Align existing library policies so that there is a unified library policy.	III
Determine if any library at any site oversees hospital archives.	III

Put backup plans in place and plan for parallel operating systems for as long as needed during the M&A process.	III
Decide when you can and will say no to providing library services at any incoming site(s).	III
Decide how work will be distributed to library team members during and following the M&A process.	III
Consider creating a plan that, over time, will allow for assignment of responsibilities to librarians by skill (systematic review, teaching, reference) rather than site, if more than one librarian is on the team.	III
After analysis, create and implement any new assignments and staff, librarian, and volunteer responsibilities.	III
After evaluating staff roles and responsibilities, determine if new services can be offered, such as healthcare system archive management, scholarly activity support, publications tracking, and others.	III
Ensure all job descriptions are updated to reflect any changes as a result of the M&A.	III
Create a unified system for in-person and virtual library education and instruction.	III
Create collaborative teams to co-teach and investigate new library resources.	III
Create a list of contacts for technical support for all e-resource platforms, hardware, and software.	III
**Budget and Finance**	
Learn about the purchasing process.	I
Learn about the procurement process.	I
Learn about budget lines and cost centers for all sites.	I
Learn about the budget structure for the newly merged system.	I
Identify the budget cycle for each site.	I
Identify which department or budget line contributes to librarian and library staff payroll.	I
Ensure payroll is processed for incoming librarians and library staff.	I
Identify the users and resources the library budget is intended to support.	I
Determine what costs can and cannot be merged.	I
Calculate cost projections.	I
Identify library sites that are earning income.	I
Identify all external funding sources (i.e. grants) for the libraries.	I
Learn about the terms and conditions of any grant funding the library receives.	I
Learn about the charge-back process for cost centers.	II

Learn about the budget structure for all sites.	II
Learn about the budget consolidation process.	II
Determine how the merger will impact internal and external funding.	II
Identify how staffing payroll contributions affect library services provided to each site.	II
Determine if funding exists to accommodate for new user groups and/or incoming sites.	II
Identify which department or budget line pays for library related costs at each incoming site.	II
Determine the factors that impact budget proposals (e.g. previous year's budget, expense history, zero-based budget, multi-year cost projections, justifications, etc.).	II
Identify contribution ratios of shared costs, frequency of contribution, and allocation of costs.	II
Determine cost allocation for incoming sites that do not have a library or library budget.	II
Seek co-purchasing agreements from incoming sites.	II
Determine whether budget consolidation is possible between all sites.	II
Determine whether budgets should be consolidated between all sites.	II
Demonstrate the cost savings that occur through centralization of library services and resources.	II
Collaborate with the procurement office if new costs are being absorbed by the library budget.	II
Consider how memberships in regional consortia, state associations, or other groups that offer bulk pricing for resources impact budgets and procurement.	II
Strategically use up any surplus of funds available in the existing budget lines.	II
Learn the payroll process.	III
Learn about the financial infrastructure of the healthcare system.	III
Learn about the budget approval process.	III
Learn about cost per use (CPU).	III
Reallocate any extra money saved as a result of the merger to pay for other library related costs.	III
Calculate income projection based on previous history, if earning income.	III
Learn about how the income offsets the expenses, if earning income.	III
**Marketing and Outreach**	
Work with the web team to create an online presence for the library if one does not exist.	I

Identify the health system's marketing and communications liaison for each site.	II
Develop and distribute a consolidated promotional handout/digital brochure highlighting library resources and services.	II
Create a standardized logo and/or message for the library.	II
Collaborate with human resources to identify how library promotional materials can be incorporated into new employee orientation or onboarding activities.	II
Arrange library orientations and instructional sessions for new user groups.	II
Utilize library instructional sessions as a way to promote other library resources and services.	II
Identify different opportunities to advocate and highlight the library's resources and services.	III
Learn about the health system's branding guidelines and ensure marketing materials align with them.	III
Identify the best method to distribute or communicate information to specific library user groups.	III
Collaborate with the health system's marketing and communications liaison to identify the methods of communication and promotion that can be used at each site (i.e. specific email distribution lists, newsletters, messaging boards, etc.).	III
Identify which librarians will be responsible for outreach activities at specific sites.	III
Create a marketing and outreach plan for all the libraries in the system.	III
Identify new and existing user groups that can be targeted for marketing campaigns	III
Create talking points that address the most frequently asked questions about the library.	III
Create marketing materials that highlight the importance and value of library services.	III
**Physical Library Spaces**	
Conduct needs assessment for library space.	II
Discuss the retention and repurposing of library space.	II
Determine what hardware is needed in any physical library space to support the library users.	II
Assess library space for all sites including potential losses or relocations.	III

LIBRARY COLLECTIONS & INFORMATION SYSTEMS

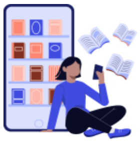

- *Information Technology & Services*
- *Vendor Relations*
- *Collection Management*

LIBRARY COLLECTIONS & INFORMATION SYSTEMS	
**Information Technology and Services**	
Identify the IT/IS representative for each site.	I
Introduce yourself to the IT/IS team that will be handling access and network configuration.	I
Identify the IT/IS unit that oversees and troubleshoots remote access.	I
Educate the IT/IS team about the library's license agreements and IP authentication needs.	I
Identify the rights and access to existing hardware, software, web-based, and cloud-based platforms for each site.	I
Ensure that incoming staff complete IT/IS onboarding (e.x. employee credentials, remote access, email activation, cloud storage access).	I
Ensure that all incoming staff are provided with the necessary hardware and equipment to facilitate their work on-site or off-site.	I
Learn about IT/IS security guidelines and policies for each site.	I
Learn about the IP address infrastructure for each site and its effect on access.	I
Learn about whether the healthcare system will use dynamic or static IP addresses and its implications on centralization of eResources.	I
Define tasks that are IT/IS's responsibilities and the library's responsibilities during the merger.	I
Identify unique user groups who have access to library eResources.	I
Identify user groups that do and do not have remote access to eResources.	I
Ensure policies and procedures are in place to address different remote access points to the library e-resources.	I
Plan for expanding subscription access to incoming site(s).	I
Assess the compatibility of the incoming library's eResources with the existing site's IT/IS infrastructure.	I
Identify authentication system to subscribed library eResources.	I
Inventory hardware (e.g. desktops, laptops, screencast technology, etc.) for all sites including equipment assigned to remote library staff.	I

Learn about IT/IS onboarding processes (e.g. employee credentials, remote access, email activation, cloud storage access).	I
Centralize authentication system to subscribed library resources.	I
Determine whether to consolidate or switch to a new discovery system.	II
Create a shared space for documents for all sites.	II
Discuss existing contracts between the IT/IS department and service providers for incoming sites (e.g. service line agreements).	II
Create one central website for library resources and services.	II
Advocate for one central website for library resources and services.	II
Determine whether IT/IS services are operated internally or by a 3rd party.	III
Discuss the timeline for changes to IP infrastructure for the newly merged system.	III
Define communication expectations relating to any changes to the IP infrastructure.	III
Identify the ILS/LMS system that is used at all sites	III
Determine whether to consolidate or switch to a new ILS/LMS system	III
Identify the discovery system used at all sites.	III
Identify which video conferencing tools (e.g. Zoom, Teams, WebEx, etc.) are used and supported.	III
Create one central system for collecting and addressing requests for library services.	III
**Vendor Relations**	
Identify library resource vendors for all library sites.	I
Create a list of who to contact to learn about the different contracts the library may manage.	I
Learn which site(s) have multi-year contracts with specific vendors	I
Learn about what hospital metrics influence the cost of library subscriptions.	I
Learn about the pricing model used by each vendor.	I
Learn about how each licensing agreement explicitly defines what a hospital, healthcare system, and a clinic are; learn how they are counted by the vendor.	I
Learn about the terminology included in multi-year licensing agreements.	I
Identify which licensing agreements and costs are duplicative across all sites.	I
Identify specific licensing agreements that may need to remain duplicative within the healthcare system for a period of time	I
Create a plan and a timeline to renegotiate licensing agreements once they all have been collected.	I

Learn about licensing agreement start dates and end dates.	I
Create a working group to identify licensing agreements to prioritize and renegotiate.	I
Determine which resources to continue or discontinue and negotiate with the appropriate vendor.	I
Prioritize contract negotiations with vendors that are shared between the health system and the incoming site(s).	I
Communicate honestly to each vendor about the new size of the health system and negotiate in good faith.	I
Create a workflow to notify vendors of expanded IP address access.	I
Communicate with vendors about the changes to IP addresses, where applicable.	I
Notify vendors about the new site(s) and/or user groups.	I
Communicate with vendors the metrics (number of beds, clinicians, hospital sites, etc.,) for the new site(s).	I
Share the addresses of all new library site(s) with the vendors.	I
Negotiate with the vendors to unify licensing agreements that the acquiring institution and new library site have in common.	I
Negotiate with vendors to eliminate duplicative licensing agreements and streamline costs.	I
Collect information about the audience, content, and format of each specific platform/resource used by the incoming site(s).	II
Assess how academic affiliations will impact licensing agreement negotiations.	II
Create a long term plan to manage and oversee renewals, renegotiations, payments and multi-year licensing agreements.	II
Work with vendors to apply the same end date to all contract renewals the acquiring institution has in common with the new library site(s).	II
Identify when it is legally acceptable to communicate with vendors about the changes to the library.	III
**Collection Management**	
Conduct system wide inventory of all the print and electronic resources held in the collection and create a master list.	I
Learn about what resources must remain accessible at specific sites.	I
Determine what resources need to be universally accessible.	I
Identify what subscriptions need urgent and immediate attention.	I
Determine how the merger will impact consortium agreements.	I

Prioritize what resources in the collection must be maintained/kept and what resources can be terminated/discarded, in the event of budgetary changes.	**II**
Gather and maintain usage statistics for print and electronic library resources for all library sites.	**II**
Move away from print to e-resources as much as it is fiscally possible.	**II**
Determine if the collection supports unique user groups.	**III**
Identify where there are gaps in the collection.	**III**
Evaluate the entire collection and determine what is needed and what is desired.	**III**
Evaluate and leverage the opportunities presented by the merger to change or expand the collection.	**III**
Review any existing collection development policies for all library sites.	**III**

LIBRARY STAFF INTEGRATION & INTERCONNECTION

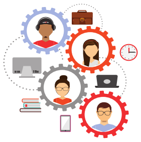

- [Library Staff Training & Development]
- [Library Staff Relationships]
- [Interpersonal Wellness]

LIBRARY STAFF INTEGRATION & INTERCONNECTION	
**Library Staff Training & Development**	
Provide incoming staff with access to the appropriate technology to participate in staff workflows (i.e., keeping statistics, library calendar access and regular meeting invitations).	I
Interview all librarians and library staff at incoming sites to understand their skills and strengths.	II
Identify which librarians and library staff need additional support and/or professional development in order to fulfill newly assigned responsibilities.	II
Create a centralized method of communication for knowledge sharing among all librarians and library staff.	II
Identify librarians and/or library staff whose positions may become redundant and offer opportunities to learn skills and training to remain part of the library team.	II
Create a standardized method to train all staff on system policies and procedures.	III
Create a standardized method to train all staff on library policies and procedures.	III
Determine what onboarding method will be used for new librarians and/or library staff.	III
Provide proper training to all staff so that they are able to fulfill assigned responsibilities.	III
**Library Staff Relationships**	
Create a centralized method of communication (i.e. email distribution list, Teams channel, slack, etc.) for all librarians and library staff.	I
Create a welcoming environment for all librarians and library staff from incoming sites.	I
Promote a workplace culture of collegiality and not competition.	I
Create opportunities for librarians and library staff at all sites to build working and social relationships with one another.	I
Find ways to include and support librarians and/or library staff who are uncooperative, stand-offish, or reluctant to collaborate.	I

Communicate honestly and forthrightly about any librarians and/or library staff whose positions have been eliminated because of the merger.	I
Learn about best practices for team-building to assist in creating working relationships for all librarians and library staff.	II
Meet every librarian and library staff member to learn about career path interests, concerns, and ideas in order to establish rapport.	II
Create channels for communication between librarians and library staff at all sites.	II
Schedule regular meetings so all librarians and library staff can meet.	II
Create opportunities (i.e. committees, working groups, projects, etc.) for librarians and library staff from incoming sites to collaborate with librarians and library staff at all other sites.	II
Support all librarians and library staff in voicing their concerns and ideas for making things work in the new environment.	II
Seek buy-in from librarians and library staff when consolidating specific departments between existing and incoming sites.	II
Seek buy-in from librarians and library staff when roles and responsibilities change between existing and incoming sites.	II
Create a shared calendar for the library(ies).	III
**Interpersonal Wellness**	
Acknowledge that the library is not central to the M&A process.	I
Strongly advocate for the library.	I
Establish realistic expectations on what can and cannot be achieved.	I
Acknowledge the M&A process may lack organization and direct leadership.	I
Acknowledge that the process of unifying into one library system may be difficult due to physical distances between sites, diversity of user group needs, and the institutional culture towards medical libraries.	I
Acknowledge that incoming sites may be resistant to contributing to the library budget, especially if the site never before provided library access and services.	I
Acknowledge that a lack of leadership by and collaboration among the librarians can cause stress and negatively impact everyone's emotional well-being.	I
Avoid trying to stay unnoticed in an effort to deter being targeted, as it may result in the library becoming a low priority during the M&A process.	I
Acknowledge that librarians or library staff may be territorial and/or mired in organizational politics.	I

Acknowledge the frustration that comes when you and others do not feel heard during the M&A process.	I
Acknowledge and address the fear of losing colleagues and becoming overworked and overloaded.	I
Establish reciprocal trust between leadership and staff.	I
Acknowledge that the libraries will most likely be understaffed and justifying the addition of staff will always be a challenge.	I
Encourage staff to use Employee Health Services, Employee Assistance Programs, Employee Wellness Initiatives, or other available assistance to address their stress and struggles with mental health and emotional well-being.	I
Prepare to take a proactive role in the M&A process if you are a solo librarian.	I
Acknowledge the feelings of fear, uncertainty, and frustration that come with the M&A experience.	II
Acknowledge the lack of trust that comes with the M&A process.	II
Recognize that the current M&A is not a threat, but rather an opportunity to grow and thrive individually and as a department.	II
Adopt a forward-thinking and positive outlook during the M&A process.	II
Recognize that you will need to be flexible and may need to pivot in order to respond to issues that arise.	II
Acknowledge the importance of funding in achieving any goals for the library during the M&A process.	II
Acknowledge that there will be different library needs based on the type of hospital and overcoming these differences will require sensitivity to the needs of the library user groups.	II
Acknowledge there will be different approaches to offering library services based on librarians’ philosophy of librarianship, and overcoming these differences will require sensitivity to the perspective of all the librarians.	II
Acknowledge that leaving libraries isolated by not collaborating across distance and keeping budgets unconsolidated is a barrier to centralization.	II
Understand that being transparent with everyone involved is the key to success in the M&A process.	II
Avoid negative leadership qualities such as overaccommodation and "people pleasing".	II
Ensure you are sensitive and considerate to how people adjust to change in different ways.	II
Reach out to colleagues and mentors throughout the M&A process for emotional support.	II

Ensure you provide an opportunity for staff and colleagues to be heard during the process.	II
Understand that if layoffs and terminations occur, positions of higher-salaried librarians are most often cut, and the loss of leadership causes a lot of stress and low morale for any librarians and library staff that remain.	II
Acknowledge there will be librarians and/or library staff who are uncooperative, stand-offish, or reluctant to collaborate.	II
Utilize opportunities for collaboration as a method to build trust among librarians and library staff at all sites.	II
Encourage sharing between all librarians and library staff to minimize territorial disputes or feelings.	II
Watch for staff that may be isolated due to physical distance or remote work arrangements.	II
Recognize the amount of time, effort, and technology needed to build a geographically dispersed team.	II
Provide opportunities for geographically dispersed team members to build working relationships in order to minimize silos by site.	II
Acknowledge that a unified or centralized team has a greater chance for success.	II
Set expectations to reflect the unification of library teams.	II
Acknowledge that you may never become a unified library team due to barriers outside your control.	II
Acknowledge that the M&A process can lead to the development of powerful emotions and possibly mental health issues for librarians and library staff.	II
Acknowledge that librarians and library staff will feel frustrated, angry, and anxious when administration creates an organizational structure that is illogical.	II
Learn about frameworks and best practices in managing change within an organization.	III
Evaluate your past experience and current situation in order to make plans for yourself and the library in the future.	III
Acknowledge the transformational nature of the change an M&A brings, and that such change takes time.	III
Hope for the best-case scenario but prepare for the worst case scenario.	III
Acknowledge the difficulty of integrating teams and that team building takes time.	III
Acknowledge that a lack of team cohesion among librarians and library staff creates low morale and can lead to high turnover.	III

Acknowledge that people will react to feeling both overworked and underworked due to reconfigurations of staff responsibilities during the M&A process.	**III**
Understand that an unfair distribution of tasks and an inequitable reconfiguration of staff responsibilities will lead to a lack of team cohesion among librarians and library staff.	**III**

HEALTHCARE SYSTEM ORGANIZATION

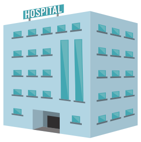

- *System Organization Structure*
- *Executive & Leadership Relations*
- *Organizational Relationships*

HEALTHCARE SYSTEM ORGANIZATION	
**System Organizational Structure**	
Determine where the library fits within the organizational structure of the health system.	I
Identify the department or unit that oversees the library for each incoming site(s).	I
Identify point people responsible for the library during the M&A process.	I
Identify the logistical structure of the purchasing department at all sites in order to determine how to make purchases.	I
Identify the logistical structure of the IT/IS department at all sites in order to determine how to request IT/IS assistance.	I
Learn about and understand the health system organizational structure.	II
Educate yourself about how each hospital site fits within the larger healthcare system as determined by the affiliation agreement.	II
Clarify the organization of and reporting structure for leadership of all sites.	II
Determine if the existing organizational structure for the libraries can be centralized under one department within the healthcare system (e.g. IT, GME, Office of Academic Affairs, Medicine, etc.)	II
Identify if a library committee or library chair exists at each site or system.	II
Identify unique service lines and the resources that are necessary to support them.	II
**Executive & Leadership Relations**	
Identify key executive leaders who are responsible for the library during the M&A process.	I
Identify expectations of the executive administration from incoming sites in regards to their financial contributions to the library budget.	I
Identify the executive administration's expectations for access to library resources and services in the newly merged system, particularly for incoming sites.	I
Educate executive administration on payment responsibilities for library resources and services.	I

Educate executive administration on expected financial impact to the library budget when expanding library access, resources and services.	I
Educate executive administration on budget eliminations that are conducive to cost savings and those that are not.	I
Educate executive administration on how elimination of library positions are not conducive to cost savings.	I
Educate executive administration about the negative effects of eliminating library budget lines for incoming sites.	I
Educate executive administration on the realistic timeline for expiration and sunset of license agreements for library resources for incoming sites.	I
Educate executive administration on the need for temporarily managing duplicate license agreements for library resources.	I
Recognize that there is a common misperception by executives at incoming sites that acquiring systems will pay for the site's library budget when that may not be the case.	I
Ensure that the incoming site does not eliminate their library budget due to the idea that the acquiring system will cover the entire library budget.	I
Determine whether library positions are going to be eliminated.	I
Advocate against minimizing the number of library positions and collaborate with executive administrators in order to retain library positions.	I
Advocate for additional funding or compensation if the library is required to expand library access, resources and services as a result of the M&A.	I
Develop a script outlining the importance and needs of the library to get continued support from executive administration.	II
Introduce yourself to executives from incoming sites when legally allowed during the M&A process.	II
Establish communication channels with key executive stakeholders involved in the M&A.	II
Meet with leadership to define the goals for the library during each phase of the M&A process.	II
Develop a relationship with key executive leaders to get buy-in and support for the library during the M&A process.	II
Identify the executive administration's vision for the library in the newly merged system.	II
Discuss anticipated changes with executive administrators at all sites.	II
Share the library's strategic plan with appropriate executive administrators and incorporate their feedback.	II
Inform executive administration about collection management plans and get their buy-in and support.	II

Champion ways for the library to have a voice in the M&A process or ideally a seat at the table throughout the M&A process.	II
Advocate to executive administration for opportunities to provide input relating to decisions on library access, resources and services.	II
Emphasize to administration that library services need to be included in the service level M&A agreements.	II
Advocate for beneficial changes to the library during the M&A process.	II
Develop a plan to get buy-in from executive administration and librarians/library staff for centralizing the library over time.	II
Advocate to executive administration for the creation of one centralized library system.	II
Advocate to executive administration that regardless of the size of the organization, libraries work better when more resources and services are centralized.	II
Advocate for shared cost of new technologies purchased after the M&A.	II
Advocate for additional library positions or increased compensation to reflect expanded responsibilities.	II
Ensure that executive administration supports professional development to support the transition to new roles and responsibilities.	II
Recognize that there may be instances when key executive leaders delay or deprioritize making decisions about the library which then causes difficulties and barriers during the M&A process.	III
Recognize that there may be instances when key executive leaders may seek greater control or authority over the library which then causes difficulties and barriers during the M&A process.	III
**Organizational Relationships**	
Develop a relationship with training program leadership at incoming sites who use library educational sessions and resources.	I
Develop a script for user groups from incoming sites who will not be receiving library access and services.	I
Create a system wide Library Integration Committee to oversee the integration of all sites during the M&A process.	I
Meet with key stakeholders in nursing, graduate medical education, continuing medical education, faculty development, and other academic departments from each incoming site.	II
Meet with physician leaders to inform them about library services and resources, and encourage them to advocate for their use.	II
Develop a relationship with nursing leaders at any site pursuing or working to maintain MAGNET status.	II

Determine where quagmires and infighting about access may arise and plan ways to avoid or address such problems.	II
Identify library supporters/champions that can advocate for access to library resources and services.	II
Clearly communicate and provide frequent updates to user groups who are receiving library access and services they previously did not have.	II
Learn why specific user groups utilize library services and resources.	III
Develop a relationship with coordinators and leaders of Nursing Residencies and Fellowships at each incoming site.	III
Clearly communicate with all user groups as early and as frequently as possible.	III
